# Rethinking the reliability and accuracy of biomarkers in CNS-originating EVs for Parkinson's disease and multiple system atrophy

**DOI:** 10.3389/fneur.2023.1192115

**Published:** 2023-09-05

**Authors:** Hash Brown Taha

**Affiliations:** Department of Integrative Biology and Physiology, University of California, Los Angeles, Los Angeles, CA, United States; Department of Integrative Physiology, University of Colorado Boulder, Boulder, CO, United States

**Keywords:** synucleinopathy, extracellular vesicles, biomarkers, differential diagnose, Parkinson's disease, L1CAM, parkinsonism, phosphorylated synuclein

Synucleinopathies are a group of neurodegenerative disorders characterized by abnormal deposition of α-synuclein (α-syn) aggregates in neuronal and glial populations. However, α-syn implicated in synucleinopathies often affects different cellular populations. For example, in Parkinson's disease (PD), α-syn accumulates in neurons leading to Lewy bodies (LB), while in multiple system atrophy (MSA), α-syn deposits as glial-cytoplasmic inclusions in oligodendrocytes and LBs in neurons (1, 2). Despite pathophysiological differences, the two diseases are often misdiagnosed due to clinical symptoms overlap, especially in the early stages, hindering appropriate enrollment and stratification in clinical trials (3, 4).

Given that both diseases can only be definitively diagnosed postmortem by a neuropathologist, there is a dire need to find a minimally invasive way to accurately diagnose both diseases antemortem. Biomarker discovery using cerebrospinal fluid (CSF) and imaging modalities such as PET, SPECT, or MRI has been the state of the art for neurodegenerative diseases, including synucleinopathies (5, 6). However, these strategies are limited by their invasive and/or expensive nature, their low diagnostic power for synucleinopathies, their lack of reproducibility among studies, and the need for a high level of expertise and/or sophisticated technologies.

Extracellular vesicles (EVs) compromise a heterogenous group of exosomes, ectosomes and apoptotic bodies. They carry proteins, carbohydrates, lipids and nucleic acids representing their cell of origin. Exosomes are the smallest and most abundant EVs, ranging in diameter between 30 and 200 nm and are thought to communicate cell-state-specific content (e.g., stimulated, differentiated, stressed) with both neighboring and distant cells (7). In synucleinopathies and other neurodegenerative proteinopathies, EVs carry pathological forms of the proteotoxic proteins, e.g., α-syn oligomers, presumably to remove them and protect the cells from further damage (8). The EVs can cross the blood-brain barrier (BBB) into the blood and protect candidate biomarkers from enzymatic degradation (9). Thus, measuring cell-state-specific biomarkers in CNS-originating EVs isolated from the blood may provide a window into the brain's biochemistry, though is far from practical applications due to technical limitations.

Several recent studies have investigated the potential of measuring biomarkers in CNS-originating EVs for the differential diagnosis of patients with PD and/or MSA, specifically using neuronal EVs (9–14), and to a lower extent oligodendroglial EVs (nEVs and oEVs, respectively) (11, 13, 15). One such study (13) found that a combination of nEVs α-syn, oEVs:nEVs α-syn, oEVs phosphorylated α-synuclein at Ser129 (pS129-α-syn) and putative “exosome” (CD81+) particle concentration may improve the separation between patients with PD and MSA compared to the previous model (11). In this opinion paper, I will expand on the study's results, offer guidelines for data interpretation, and suggest future steps.

In the previous study, the authors aimed to measure levels of nEVs and oEVs pS129-α-syn in 32 healthy controls (HC), 46 PD, and 30 MSA samples as well as nEVs and oEVs tau in 54 HCs, 51 PD and 41 MSA samples (13) using samples obtained previously (11). LBs and GCIs are highly enriched in pS129-α-syn, making it a promising biomarker for synucleinopathies (16), while tau is associated with PD (17) but is rare in MSA (18). The findings (13) suggested that patients with PD and MSA may have higher levels of pS129-α-syn in oEVs in comparison to HCs, while patients with MSA may have lower tau levels in nEVs and oEVs in comparison to patients with PD and HCs.

Importantly, oEVs pS129-α-syn did not significantly differ between patients with PD and MSA. Due to poor overlap between the pS129-α-syn and tau measurements in the same subset of samples, the authors conducted receiver operating characteristic (ROC) models separately for each group. A multinomial logistic regression with LASSO variable selection selected nEVs α-syn, oEVs:nEVs α-syn, oEVs pS129-α-syn and CD81+ particle concentration to establish the discriminative ROC model for the former group. The ROC model separated PD from MSA with AUC = 0.936. In contrast, the model encompassing nEVs α-syn, oEVs:nEVs α-syn, oEVs tau and CD81+ particle concentration did not improve the separation.

While these results may seem promising, they are limited by various factors (summarized in Figure 1). The results from the previous study (13) showed high overlap in the data, as can be seen in the error bars in Figures 1A–C (13), with an approximately equal number of samples, which suggests that the results may not be significant (19). Further, in another subset of samples, measured ~2.5 years later, pS129-α-syn in CNS-originating EV lysates resulted in signals below the lower limit of detection (LLoD) of the assay. This may suggest that the protein had deteriorated or aggregated over time. It may also suggest that batch-to-batch variability due to the 2.5-year gap between measurements led to confounding results as suggested by Lashuel et al. (20).

**Figure 1:**
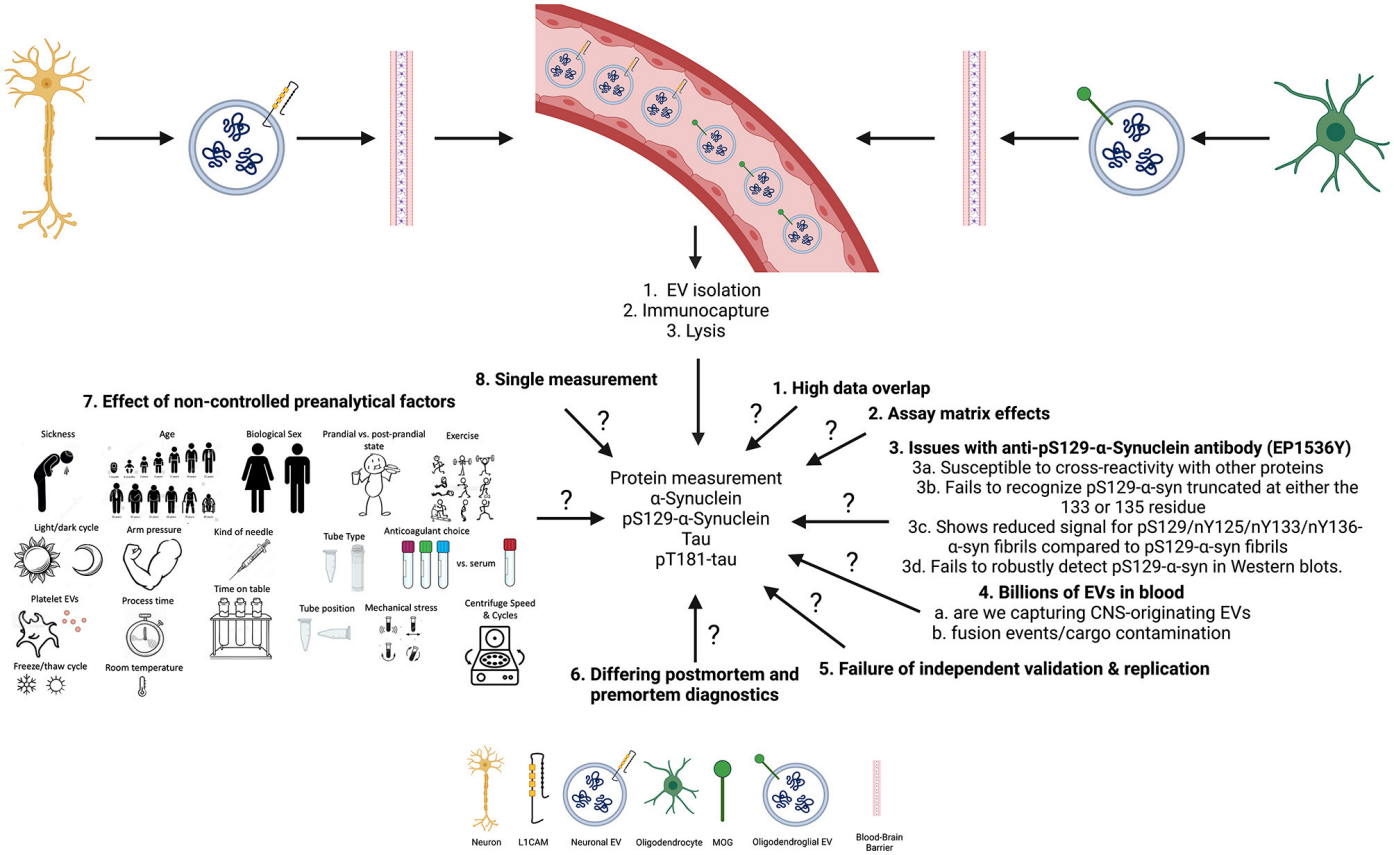
Summary of open questions and key limitations that may impact the measurements of biomarkers in CNS-originating extracellular vesicles (EVs).

The pS129-α-syn measurements were conducted using an in-house electrochemiluminescence ELISA (ECLIA) with an antibody highly specific for pS129-α-syn (EP1536Y) for capture and the SULFO-TAG anti-α-syn antibody provided by Meso Scale Discovery for detection. However, looking at Figure 3 (21), the data showed poor dilution linearity and spike recovery results, indicating that the levels of pS129-α-syn in patients with PD and MSA or HCs found in our study may have been due to a matrix effect. It is also important to note that the authors conducted the dilution linearity and spike experiments in a non-traditional way: the samples were not spiked above the ULOQ or diluted sufficiently for dilution linearity or spiking experiments, respectively.

Although, the authors showed that the anti-pS129-α-syn EP1536Y antibody is highly specific for pS129-α-syn (as seen in their Figure 1) (21), these findings suggest caution is needed when interpreting the results from the ECLIA assay which may work in serum samples only with the possibility of confounding matrix effects, but not plasma or CSF.

Though pS129-α-syn is commonly targeted as a marker of synucleinopathies, α-syn is also prone to concomitant posttranslational modifications and C-terminal truncations (20, 22). This led the group of Lashuel et al. (20) to investigate how these issues may impact various antibodies specific for pS129-α-syn. Their findings suggest that the EP1536Y antibody is susceptible to cross-reactivity with other proteins, fails to recognize pS129-α-syn truncated at either the 133 or 135 residues, shows reduced signal for pS129/nY125/nY133/nY136-α-syn fibrils compared to pS129-α-syn fibrils, and fails to robustly detect pS129-α-syn in Western blots. These findings suggest that the results seen with nEVs and oEVs pS129-α-syn in patients with PD, MSA, and HC are not reliable until further characterization is done. Moreover, this particular antibody may react with the antibodies used for capturing nEVs and oEVs as seen with the α-syn antibody (23).

The authors used a two-step procedure to capture CNS-originating EVs, first by using a polymer-based precipitation technique (i.e., ExoQuick) followed by immunocapture of nEVs or oEVs using the anti-L1CAM or the anti-MOG antibody, respectively. Notably, polymer-based precipitation techniques offer poor reliability, provide a heterogeneous mixture of particles, aggregated proteins and salts, and are expensive. Therefore one may wonder whether the measured analytes do indeed originate from EVs. Furthermore, the authors had not characterized pS129-α-syn levels directly in the serum/plasma or directly in the bulk EVs before immunoprecipitation of nEVs or oEVs, limiting the interpretation of the results. Lastly, it is unclear why the LASSO model selected oEVs pS129-α-syn to improve the separation among the groups, especially patients with PD and MSA, given that it was not significantly different between the two diseases (*p* = 0.52), which is supported by a meta-analysis (24).

On the other hand, tau has been linked to PD pathology. Thus, the authors have attempted to measure nEVs and oEVs tau, and the findings did not suggest improved separation among the groups with high overlap and many samples being below the LLOD of the assay. As this is a commercial assay, the authors did not further characterize it in the published work. Similar to the above, the authors did not measure tau directly in serum/plasma or in bulk EVs before immunoprecipitation of nEVs or oEVs (13). Furthermore, the authors obtained no signals for the measurement of pT181-tau in the majority of nEVs and oEVs of patients with PD, MSA and HCs as well as another subset of patients with tauopathies.

Another critical point that the authors did not address is the control of the preanalytical variables and how they may impact the purity, property, number and content of EVs isolated. Many studies have shown that preanalytical variations such as the time from blood collection to the first centrifugation at various temperatures, centrifugation force and time, depletion of platelets, the number of freeze/thaw cycles before EV isolation, choice of anticoagulation agent mixed with plasma, the time of preparation, centrifugation methodology, the nature of transport, number of freeze/thaw cycles, storage conditions, temperature and the type of collection tube have all been shown to influence the purity, property, number and content of EVs isolated (25, 26).

None of these variables have been controlled in either Dutta et al. (14) or Taha et al. (24), further indicating that the results might be due to chance which explains (see below) why other groups have failed to replicate these findings. Specifically, in Taha et al. (13) the authors did not perform subgroup analyses by repository, even though different biobank repositories do indeed have different protocols. It is also important to note that the technique used here is of high cost and requires approximately 3 days to obtain lysates of CNS-originating EVs from the blood.

Current efforts by ISEV (27) and others (28, 29) are aiming toward more rigorous standardization so that findings in EVs can be replicated successfully. All studies measuring biomarkers in CNS-originating EVs for parkinsonian disorders (9, 10, 12, 14, 15, 30–35) are encouraged to keep detailed record of their methodology and handling steps through EV-TRACK (36).

Finally, there are three more critical points to take into consideration when interpreting the results. First, other groups have tried to replicate the previous findings (11, 13) and were not successful. Second, few of the patients included in the original sample cohort have passed away, and neuropathological diagnosis showed a different diagnosis from the one obtained premortem. Third, the findings in the latest study (13) have not been validated in an independent cohort while no study to date replicated the findings in the original study (11), further weakening the appropriate interpretation of the results.

## Conclusion

It is my opinion that caution should be exercised when interpreting the results from the previous two studies (11, 13) that use CNS-originating EVs for the differential diagnosis of patients with PD and MSA. Despite the potential benefits of measuring cell-state-specific biomarkers in CNS-originating EVs, there are several limitations to this approach that must be considered. The high overlap in data, the issues with the poor characterization of the developed pS129-α-syn assay due to the possibility of confounding matrix effects as well as the problems described by Lashuel et al. (20) with the used EP1536Y antibody for capture, failure of replication by other groups, differences in diagnosis postmortem from those obtained premortem, and lack of validation in independent cohorts, suggest that these biomarkers and particular approach may not be reliable. Additionally, the lack of control for all preanalytical variables that have been shown to severely affect the reproducibility of findings with EVs suggests that these findings may be due to chance.

Therefore, more research is needed to better understand the limitations of these biomarkers in CNS-originating EVs and to identify new and more reliable markers for the differential diagnosis of synucleinopathies. It is of utmost importance that healthcare professionals thoroughly comprehend these limitations. Under no circumstances should they employ such unestablished or unlicensed tests in a clinical environment to make diagnoses for their patients.

## Author contributions

HT: literature search, conception, and writing of manuscript.
